# Association between METS-IR and female infertility: a cross-sectional study of NHANES 2013–2018

**DOI:** 10.3389/fnut.2025.1549525

**Published:** 2025-02-28

**Authors:** Haiyan Li, Hongxia Tan, Zhenbo OuYang, Xianyue Hu, Yanjing Bao, Tianyang Gao, Wenfeng Hua

**Affiliations:** ^1^Department of Reproductive Medicine Center, The Affiliated Guangdong Second Provincial General Hospital of Jinan University, Guangzhou, China; ^2^Department of Gynecology, The Affiliated Guangdong Second Provincial General Hospital of Jinan University, Guangzhou, China; ^3^The Second School of Clinical Medicine, Southern Medical University, Guangzhou, China; ^4^Research Institute for Maternal and Child Health, The Affiliated Guangdong Second Provincial General Hospital of Jinan University, Guangzhou, China

**Keywords:** insulin resistance, metabolic syndrome, metabolic score for insulin resistance, female infertility, NHANES

## Abstract

**Background:**

Obesity and metabolic syndrome are significant contributors to infertility in women and are closely associated with insulin resistance (IR). The metabolic score for insulin resistance (METS-IR) is a new, non-insulin-based fasting index used to measure IR. However, the potential of METS-IR as a predictive indicator of female infertility risk has not been established. This study aimed to explore the association between METS-IR and the risk of female infertility.

**Methods:**

This cross-sectional study used data from the National Health and Nutrition Examination Survey (NHANES) from 2013 to 2018. We conducted multivariate logistic regression, restricted cubic spline (RCS), and threshold effect analyses to investigate the relationship between METS-IR and female infertility.

**Results:**

According to the self-reported data, 188 (12.20%) participants were classified as infertile. A significantly higher proportion of participants with elevated METS-IR were found to have infertility. Multivariable logistic regression analysis revealed that METS-IR was significantly associated with increased risk of female infertility, irrespective of the independent variable analysis by continuous variables or tertiles in the fully adjusted model (Model 3, continuous variable: OR = 1.02, 95% confidence interval (CI):1.01–1.04, *p* = 0.005; tertile 3 vs. tertile 1: OR = 2.00, 95% CI = 1.21–3.28, *p* = 0.0128, p for trend =0.0126). RCS analysis indicated a linear correlation between METS-IR and the risk of infertility (*p* = 0.121), and threshold effect analysis further supported this linear association (*p* = 0.136). Moreover, above the inflection point of 32.94, the risk of infertility significantly increased with increasing METS-IR level (*p* < 0.0001).

**Conclusion:**

Our results suggest that high levels of the METS-IR index are positively associated with infertility among reproductive-aged females in the United States.

## Introduction

Infertility is defined as the inability of women to conceive after at least 1 year of regular unprotected sexual intercourse (1, 2). It represents a state of reduced fertility that affects both male and female reproductive health and has become a significant global public health issue (1). Epidemiological studies have shown that approximately 8–12% of couples of reproductive age face challenges in conceiving, with a significant proportion of cases attributed to female-specific factors, presenting major challenges to the field of reproductive health (3). Infertility has emerged as a critical issue that affects global human development. Hence, the Centers for Disease Control and Prevention (CDC) in the United States recommends prioritizing the diagnosis and treatment of infertility (4).

Epidemiological studies have suggested that infertility is a reproductive disorder caused by multiple factors, with advanced age being one of the most significant adverse factors affecting female fertility (1). Moreover, lifestyle factors, including diet, physical activity, psychological stress, smoking, alcohol consumption, and environmental exposures, such as radiation and chemical agents, are increasingly identified as significant contributors to infertility (5–8). Additionally, abnormalities in glucose levels and metabolic disorders, including obesity and metabolic syndrome, are frequently observed in infertile women (9–12). Insulin resistance (IR) reduces the sensitivity of muscle and fatty tissues to insulin and diminishes the sensitivity of the liver to suppress hepatic glucose production and output (13). IR can cause endocrine disturbances that affect follicular development, oocyte quality, and ovulatory patterns. These findings highlight the critical role of IR in infertility (14). IR is closely associated with polycystic ovary syndrome (PCOS), which is a common cause of infertility in women. Therefore, PCOS has garnered significant attention in the context of the association between IR and infertility. However, several studies have confirmed that IR is a distinct factor in infertile women and is potentially independent of PCOS (14–16).

The euglycemic-hyperinsulinemic clamp (EHC) is the gold standard for assessing peripheral tissue insulin sensitivity; however, its clinical application is limited owing to its complexity, invasiveness, and time requirements (17). Similarly, the Homeostatic Model Assessment of Insulin Resistance (HOMA-IR) and Quantitative Insulin Sensitivity Check Index (QUICKI) are commonly used in clinical settings. However, both methods depend on insulin measurements, making them less suitable for large-scale screening. Additionally, the techniques used for insulin assays can limit their accuracy and applicability, particularly in individuals with impaired *β*-cell function or those undergoing insulin therapy (18, 19). To overcome these issues, researchers have devised alternative fasting IR indices that do not depend on insulin measurements. These include the triglyceride-glucose index (TyG), triglyceride-glucose body mass index (TyG-BMI), and triglyceride to HDL-c ratio (TG/HDL-c ratio), which utilize fasting triglycerides, glucose, and lipoprotein measurements (13, 20, 21). Nevertheless, these indices, not insulin-based, have limitations in identifying IR associated with the pathophysiological aspects of metabolic syndrome (22). Metabolic syndrome is a cluster of pathological conditions characterized by IR, central obesity, high blood pressure, and elevated lipid levels. It significantly impairs female reproductive function, and metabolic disturbances can lead to abnormal regulatory mechanisms such as ovarian irregularities, hormone imbalances, and gonadal dysfunction in women, potentially increasing the likelihood of infertility (14, 23–25).

Recently, the metabolic score for insulin resistance (METS-IR) has emerged as a promising indirect method for detecting IR that correlates with the pathophysiological components of metabolic syndrome, garnering considerable interest among researchers and clinicians. METS-IR assesses IR using BMI, triglycerides, and fasting blood glucose instead of direct insulin measurements, making it ideal for large-scale screening and clinical practice (22). Numerous studies have demonstrated that METS-IR is an independent risk factor associated with several health issues, including type 2 diabetes mellitus (T2DM), kidney stones, frailty, psoriasis, and cardiovascular disease (26–30). Notably, Uysal et al. (31) identified a significant positive correlation between METS-IR and PCOS, suggesting its potential as a valuable index for comprehensive metabolic assessment of women with PCOS.

IR not only contributes to the development of various metabolic disorders but also affects female reproductive health and is significantly associated with infertility in women. To our knowledge, no studies have examined the potential association between METS-IR and female infertility. Therefore, in this cross-sectional study, we aimed to investigate the relationship between METS-IR and female infertility using a nationally representative sample of reproductive-aged women from the National Health and Nutrition Examination Survey (NHANES).

## Methods

### Data source

This study utilized data from NHANES, a comprehensive program that assesses the health and nutrition of the U.S. population, conducted by the NCHS. The survey implemented a sophisticated, multi-stage probability sampling strategy to ensure a representative sample of non-institutionalized individuals nationwide. Participants conducted household interviews to collect information on their health, socioeconomic conditions, and other pertinent factors. Physical examination and laboratory tests were conducted in mobile examination units.

The NCHS Ethics Review Committee conducted annual reviews and standardized the study protocols under Protocol #2011–17. All participants provided informed consent prior to data collection. Further information can be found at http://www.cdc.gov/nchs/nhanes/index.htm. Given that the NHANES data are publicly available, this study was exempt from the ethical approval and informed consent requirements. This cross-sectional study complied with the guidelines for improved reporting of epidemiological observational studies (32).

### Study population

**Figure 1:**
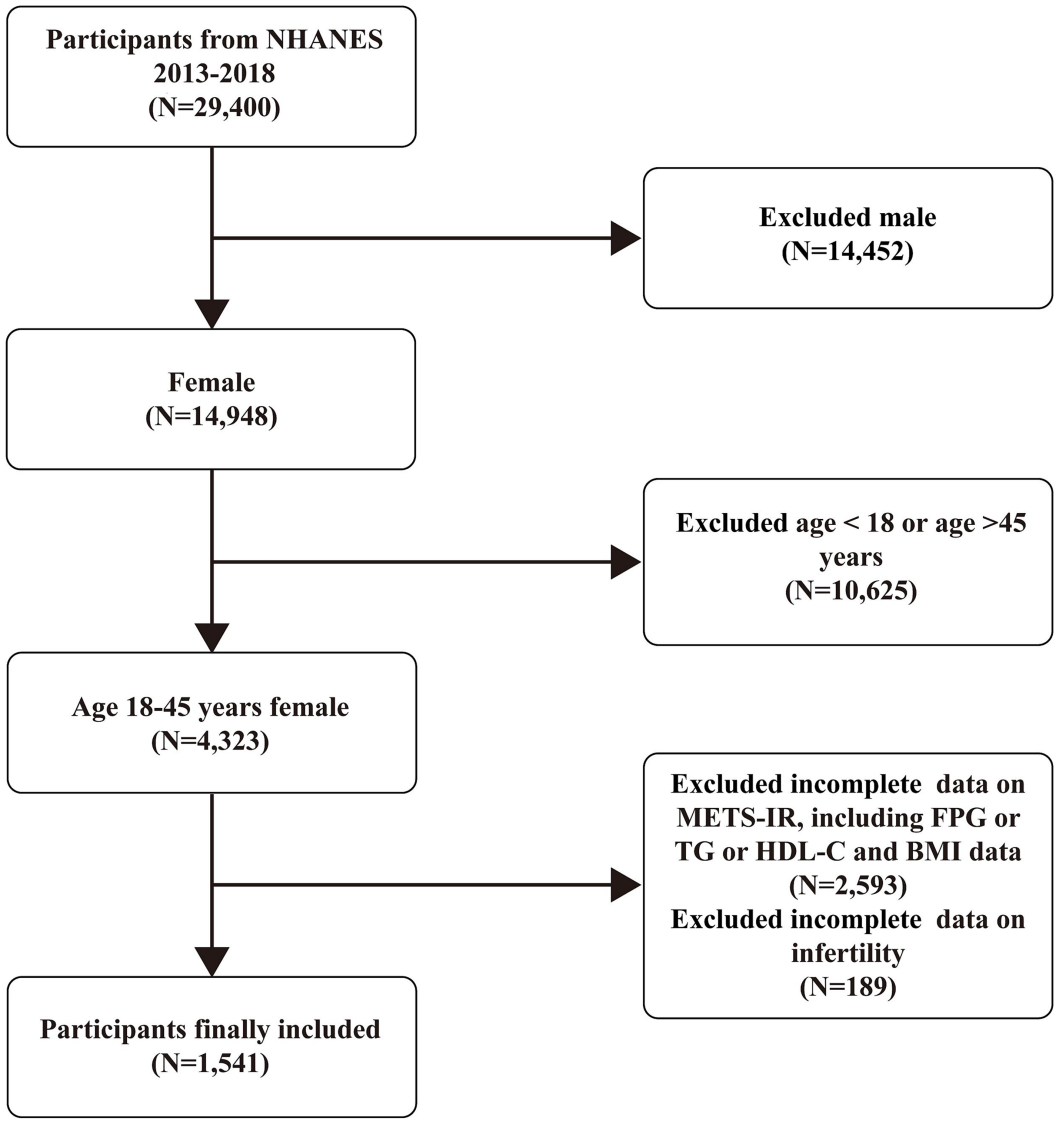
Flowchart of the inclusion and exclusion criteria.

This study analyzed data on infertility-related health issues from NHANES cycles conducted between 2013 and 2018. The analysis included participants with complete information on infertility and the METS-IR scores. Initially, 29,400 participants were included in this study. However, we excluded male participants (14,452), those aged over 45 or under 18 years (10,625), and those with missing data on METS-IR (2,593) or infertility (189). The final analysis included 1,541 eligible participants (Figure 1).

### Definitions of METS-IR and infertility

METS-IR was calculated as ln [(2 × fasting plasma glucose (FPG) (mg/dL) + fasting triglyceride (TG) (mg/dL)] × body mass index (BMI) (kg/m^2^))/ ln [high-density lipoprotein cholesterol (HDL-c) (mg/dL)] (22). All participants were categorized into three groups based on the tertiles of METS-IR: Tertile1 (19.73–32.78), Tertile2 (32.78–45.97), and Tertile3 (45.97–107.00). Quality control for laboratory tests is available at https://wwwn.cdc.gov/nchs/nhanes/Default.aspx. Infertility, the dependent variable, was assessed using questions from the Reproductive Health Questionnaire: “Have you ever attempted to become pregnant for at least 1 year without success?” (RHQ074) and “Have you ever consulted a doctor or other medical provider due to an inability to become pregnant?” (RHQ076). Participants who responded “yes” to either question were categorized as having infertility; those who responded “no” were categorized as not having infertility.

### Covariates

The covariates included in the study were age, race, marital status, education level, poverty-income ratio (PIR), alcohol consumption status (yes/no), hypertension (yes/no), diabetes (yes/no), dyslipidemia (yes/no), age at menarche, history of pelvic infection or pelvic inflammatory disease (PID) (yes/no), contraceptive pill use (yes/no), female hormone use (yes/no), and smoking status (yes/no). For detailed information on the collection of these covariates, please refer to the official NHANES website.

### Statistical analysis

All statistical analyses were conducted considering the complex, multistage clustered sampling design and appropriate NHANES sampling weights, according to the CDC guidelines. In the descriptive analysis, we compared the baseline characteristics of the participants based on their infertility status and METS-IR tertiles. Continuous variables are presented as means with 95% confidence intervals (CIs), while categorical variables are presented as percentages with 95% CIs. The baseline characteristics of the study population were assessed using weighted linear regression and chi-squared tests.

To handle missing data, continuous variables were imputed using either medians or means based on their distribution, and categorical variables were imputed using mode. We then applied weighted multivariate logistic regression models adjusted for known or potential confounders to explore the relationship between METS-IR and infertility risk. The potential nonlinear relationships between METS-IR and infertility were further evaluated using restricted cubic spline (RCS) curves based on multivariate logistic regression and threshold effect analysis. A recursive algorithm was employed to identify the inflection points, and a two-segment linear regression model was applied on either side of these inflection points. Additionally, subgroup analyses were conducted to examine the relationship between METS-IR and infertility according to age, BMI, educational level, smoking status, drinking status, hypertension, dyslipidemia, and diabetes. Statistical analyses were performed using R software, EmpowerStats, and Free Statistics. Statistical significance was set at *p* < 0.05.

## Results

### Baseline characteristics

In total, 29,400 participants were included in this study (Figure 1). Supplementary Table S1 compares the baseline characteristics of the infertile and non-infertile participants. Among them, 188 (12.20%) were categorized as infertile. Compared to non-infertile individuals, those in the infertile group were more likely to be older, consume alcohol, and have conditions such as hypertension, dyslipidemia, and diabetes. Additionally, there were differences in marital status, history of PID, use of contraceptive pills, and body mass index (BMI) between the two groups (all *p* < 0.05). Notably, the infertility group showed significantly higher METS-IR than the non-infertility group (46.87 vs. 41.22, *p* = 0.0005). This finding indicates that METS-IR may serve as a potential predictor of female infertility risk.

Subsequently, the study examined the clinical features of the participants by dividing them into tertiles based on their METS-IR scores. Compared with individuals in the lower METS-IR tertiles, those in the higher group were more likely to be older, of Mexican American descent, Non-Hispanic Black, and exhibit lower levels of education, HDL cholesterol, PIR, age at menarche, and higher levels of fasting blood glucose, triglycerides, BMI, smoking, hypertension, diabetes, and dyslipidemia (all *p* < 0.05, Table 1). Additionally, the percentage of participants experiencing infertility increased significantly from low to high (*p* = 0.0001; Table 1), with a notably higher rate in tertile 3 (20.52%) than in tertile1 and tertile 2 (10.85 and 10.18%, respectively). These differences indicate that the potential connection between METS-IR levels and infertility merits further investigation.

**Table 1:** Basic characteristics of the study population according to the METS-IR tertiles*.

	Tertile 1 (19.73–32.78)	Tertile 2 (32.78–45.97)	Tertile 3 (45.97–107.00)	*p*-value
Age(years)	29.41(28.48,30.34)	32.25(31.38,33.12)	32.59(31.96,33.22)	<0.0001
Race (%)				<0.0001
Mexican American	6.28 (4.12,9.46)	14.65(10.92,19.38)	14.92(10.93,20.03)	
Other Hispanic	8.46 (5.67,12.43)	7.21(4.81,10.67)	7.68 (5.73,10.21)	
Non-Hispanic White	61.29(53.40,68.64)	54.61(48.72 0.60.38)	52.37(45.96,58.71)	
Non-Hispanic Black	9.60(6.71,13.56)	13.22(10.17,17.01)	16.30(12.40,21.14)	
Other Race—Including Multi-Racial	14.37(10.95,18.62)	10.31(7.70,13.67)	8.73 (5.83,12.86)	
Marital status (%)				0.0055
Married	48.67(43.93,53.43)	47.30(40.88,53.81)	46.15 (41.06,51.32)	
Widowed	0.45 (0.15,1.36)	0.14(0.02,1.00)	0.41 (0.13,1.35)	
Divorced	4.39(2.44,7.77)	10.72 (7.47,15.15)	5.63(3.90,8.06)	
Separated	1.50(0.70,3.16)	2.70(1.47,4.90)	4.12 (2.62,6.42)	
Never married	31.99(26.30,38.26)	23.22(18.54,28.68)	29.04(23.64,35.10)	
Living with partner	13.01(9.59,17.41)	15.93(12.18,20.56)	14.65(11.18,18.96)	
Education level (%)				0.0204
Less than high school	10.81(7.42,15.48)	14.02(10.48,18.51)	15.46(12.90,18.42)	
High school or equivalent	17.15(13.80,21.12)	22.98(16.27,31.41)	25.18(20.93,29.97)	
College or above	72.04(66.20,77.22)	63.01(54.76,70.56)	59.36(54.70,63.85)	
Fasting blood glucose (mg/dL)	91.73 (91.06,92.40)	95.31(94.11,96.52)	107.99(105.53,110.44)	<0.0001
HDL (mg/dL)	68.88(67.13,70.64)	57.30(55.67,58.93)	46.82(45.65,47.99)	<0.0001
Triglyceride (mg/dL)	62.19(59.20,65.19)	90.08(83.47.96.69)	128.64(110.73,146.56)	<0.0001
BMI	21.63(21.38,21.87)	27.91(27.61,28.21)	38.85(38.19,39.51)	<0.0001
Family PIR	2.98 (2.77,3.19)	2.62(2.43,2.82)	2.12(1.96,2.28)	<0.0001
Smoking status (%)				0.0199
Yes	27.03(21.74,33.06)	31.18(26.36,36.45)	36.89(31.19,42.98)	
No	72.97(66.94,78.26)	68.82(63.55,73.64)	63.11(57.02,68.81)	
Drinking status (%)				0.6502
Yes	7.03 (4.80,10.19)	6.07(3.97,9.16)	7.91 (5.34,11.56)	
No	92.97(89.81,95.20)	93.93(90.84,96.03)	92.09(88.44,94.66)	
Hypertension (%)				<0.0001
Yes	5.10(3.48,7.42)	15.67(11.89,20.37)	25.99(20.90,31.82)	
No	94.90(92.58,96.52)	84.33(79.63,88.11)	74.01(68.18,79.10)	
Diabetes (%)				<0.0001
Yes	0.34 (0.08,1.40)	3.20(2.09,4.87)	13.18(10.31,16.69)	
No	99.66(98.60,99.92)	96.80(95.13,97.91)	86.82(83.31,89.69)	
Dyslipidemia (%)				0.0002
Yes	10.18 (7.18,14.23)	15.27(11.64,19.79)	22.78(18.39,27.86)	
No	89.82(85.77,92.82)	84.73(80.21,88.36)	77.22(72.14,81.61)	
Menarche (years)	13.00(12.79,13.20)	12.52 (12.37,12.68)	12.11 (11.92,12.31)	<0.0001
PID (%)				0.2448
Yes	2.86(1.58,5.12)	5.06(3.14,8.05)	4.91(2.74,8.64)	
No	97.14(94.88,98.42)	94.94(91.95,96.86)	95.09(91.36,97.26)	
Birth control pills (%)				0.4878
Yes	71.60(66.33,76.34)	73.37(68.96,77.36)	75.27(71.01,79.08)	
No	28.40(23.66,33.67)	26.63(22.64,31.04)	24.73(20.92,28.99)	
Female hormones (%)				0.1716
Yes	2.87(1.47,5.52)	5.29(3.45,8.01)	6.25(3.65,10.49)	
No	97.13 (94.48,98.53)	94.71(91.99,96.55)	93.75(89.51,96.35)	
Infertility (%)				0.0001
Yes	10.85(8.03,14.50)	10.18 (7.31 0.14.00)	20.52(16.15,25.71)	
No	89.15(85.50,91.97)	89.82(86.00,92.69)	79.48(74.29,83.85)	

PIR, poverty impact ratio; PID, pelvic infection/inflammatory disease; METS-IR, metabolic score for insulin resistance. *Percentage estimates are nationally representative using survey weights.

### Association between METS-IR and infertility

A weighted logistic regression analysis explored the relationship between METS-IR levels and female infertility. Across all three statistical models, the analysis consistently showed a positive association between METS-IR levels and infertility risk. In the fully adjusted model, each unit increase in METS-IR was associated with a 2% increase in the risk of infertility (Model 3: OR = 1.02, 95% CI: 1.01–1.04, *p* = 0.0050; Table 2). However, when METS-IR levels were categorized into tertiles, the risk of infertility in the highest tertile was significantly higher than that in the lowest tertile in Model 3, with a 100% increase in the risk of infertility (OR = 2.0, 95% CI: 1.21–3.28, *p* = 0.0128; Table 2). Additionally, trend tests across all models showed statistical significance, further supporting the significant association between higher METS-IR levels and an increased risk of infertility.

**Table 2:** Association between METS-IR and female infertility.

Exposure	Model 1 OR (95% CI), *p* value	Model 2 OR (95% CI), *p* value	Model 3 OR (95% CI), *p* value
METS-IR (continuous)	1.02 (1.01, 1.04) 0.0002	1.02 (1.01, 1.03) 0.0033	1.02 (1.01, 1.04) 0.0050
METS-IR (categorical)			
Tertile 1	Reference	Reference	Reference
Tertile 2	0.93 (0.60, 1.45) 0.7514	0.81 (0.51, 1.29) 0.3856	0.81 (0.52, 1.27) 0.3727
Tertile 3	2.12 (1.41, 3.20) 0.0009	1.89 (1.19, 2.98) 0.0098	2.00 (1.21, 3.28) 0.0128
p for trend	1.51 (1.20, 1.91) 0.0012	1.44 (1.11, 1.86) 0.0084	1.47 (1.11, 1.94) 0.0126

Model 1: Non-adjusted. Model 2: Adjusted for age and ethnicity. Model 3: Further adjusted for education level, PIR, marital status, drinking, blood cotinine, dyslipidemia, diabetes, hypertension, menstrual status, PID, birth control pills, and female.

### RCS and threshold analysis of the METS-IR and infertility correlation

**Figure 2:**
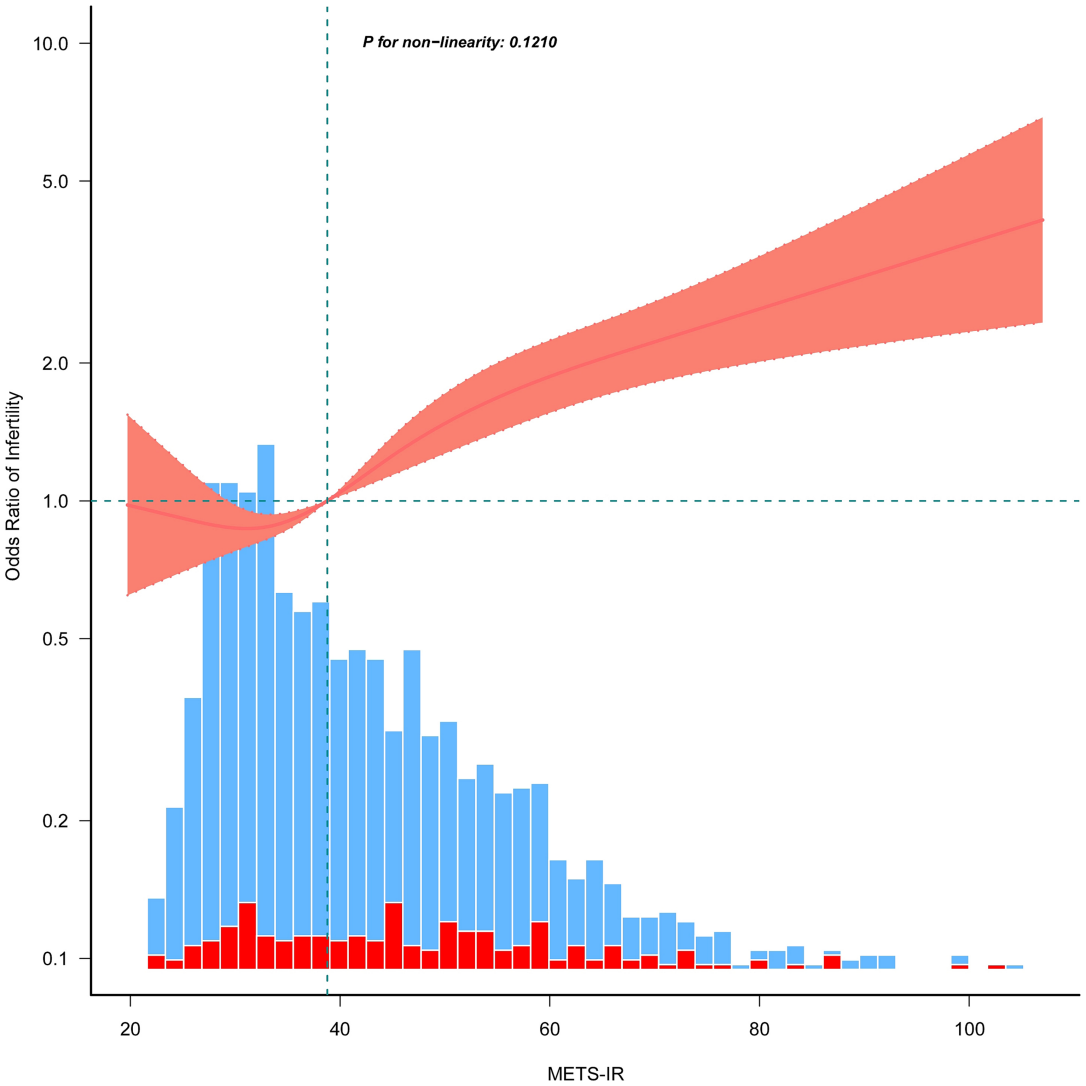
Restricted cubic spline plots for the association between METS-IR and female infertility after covariate adjustment. The red and blue histograms illustrate the percentage distributions of infertility and non-infertility cases within the study group at the corresponding METS-IR values. Thick lines in the center depict the calculated adjusted odds ratios, while the surrounding shaded areas indicate 95% confidence intervals. These calculations were adjusted in accordance with Model 3, as presented in Table 2.

To better understand the relationship between METS-IR and infertility, we conducted RCS analysis using Model 3. The results indicated a positive linear relationship and dose–response effect between METS-IR and risk of infertility (p for nonlinearity = 0.121, Figure 2). In addition, we performed a threshold effect analysis using a weighted two-stage linear regression model and regression algorithm to analyze this relationship in more detail. The results showed that the calculated inflection point was 32.94, and the log-likelihood ratio test *p*-value was 0.136, indicating that there was indeed a linear relationship between METS-IR and infertility risk. Interestingly, the results also showed that above this threshold, the risk of infertility increased by 2% for every unit increase in METS-IR values (>32.94: OR = 1.02, 95% CI: 1.02–1.03, *p* < 0.0001; Table 3).

**Table 3:** Threshold effect analysis of METS-IR and female infertility.

Infertility	OR (95% CI)	p-value
NHHR		
Model I	1.02 (1.02, 1.03)	<0.0001
Model II		
Inflection point (K)	32.94	
< K point effect 1	1.00 (0.97,1.03)	0.8803
> K point effect 2	1.02 (1.02,1.03)	<0.0001
Effect 2 minus effect1	1.03 (0.99,1.06)	0.1319
Predicted value of the equation at the folding point	−2.28 (−2.40, −2.16)	
Log-likelihood ratio test		0.1360

Adjusted for age, ethnicity, education level, PIR, marital status, drinking, smoking, dyslipidemia, diabetes, hypertension, menstrual status, pelvic inflammatory disease, birth control pills, and female hormones.

### Subgroup analysis

**Figure 3:**
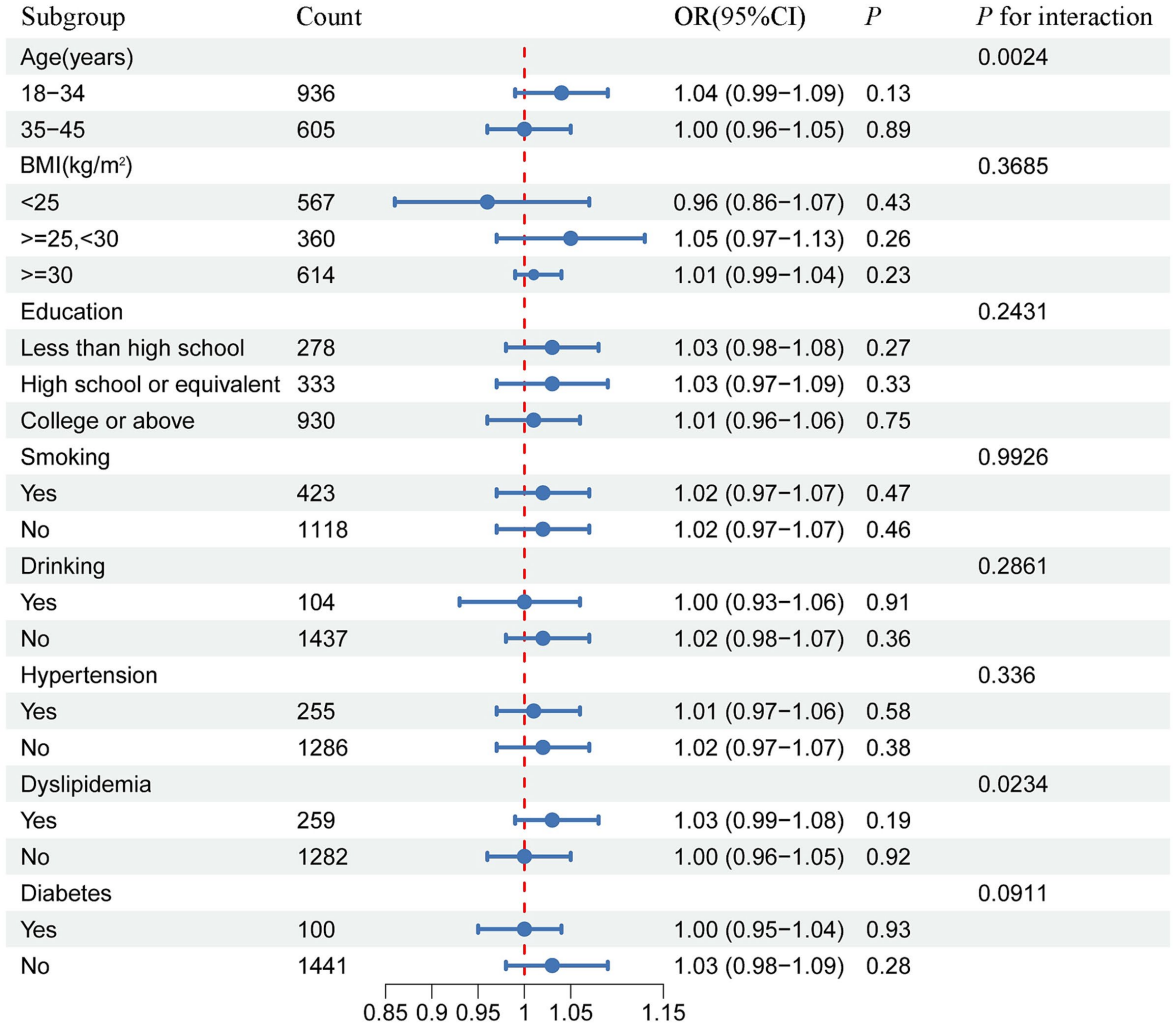
Forest plot of the stratified analysis and interaction effects on the association between METS-IR and female infertility.

Subsequently, a subgroup analysis with interaction testing was conducted to assess whether the relationship between METS-IR and infertility risk remained consistent across the different demographic groups. In Model 3, all covariates were included in the analysis, except those used to define the subgroups. The results showed a significant interaction between METS-IR and age as well as dyslipidemia (p for interaction <0.05), indicating a potential modifying effect of these factors on the association. However, no significant interaction was found between METS-IR and BMI, education level, alcohol consumption, smoking status, hypertension, or diabetes (Figure 3).

## Discussion

This large-scale cross-sectional study demonstrated that, after adjusting for potential confounding factors, METS-IR was positively associated with the risk of infertility. RCS and threshold effect analyses revealed a positive linear correlation between the METS-IR scores and infertility risk. Subgroup and interaction analyses showed that this association was influenced by age and dyslipidemia.

Obesity is closely linked to glucose intolerance and IR, both of which can negatively affect fertility and pregnancy outcomes (33). An increasing number of studies have indicated that IR and glucose intolerance are common underlying factors of obesity and PCOS. Additionally, PCOS exhibits several metabolic abnormalities that are commonly associated with obesity-related metabolic syndromes. Notably, IR is widely recognized as a key pathophysiological factor contributing to infertility in women with or without PCOS (33–35). A prospective cohort study conducted in China demonstrated that IR is associated with a reduced proportion of mature oocytes and lower embryo quality in lean infertile women without PCOS (16). Similarly, a retrospective analysis by Song et al., which involved 329 women undergoing *in vitro* fertilization, found that elevated Homeostasis Model Assessment of Insulin Resistance (HOMA-IR, an index of insulin resistance) and BMI significantly reduced clinical pregnancy rates, regardless of whether the women had PCOS (36).

Female reproductive function is negatively affected by IR through several pathways. One key mechanism involves the deterioration of oocyte quality due to IR disruption of the mitochondrial processes. Mitochondria play a crucial role as the main energy provider and a significant source of reactive oxygen species (ROS) in the oocyte cytoplasm, which is intimately connected to oocyte quality. OU et al. used a mouse model and revealed that maternal IR increased oxidative stress and disrupted mitochondrial function in mouse oocytes (37). Mitochondrial dysfunction leads to excessive ROS production, which triggers the release of inflammatory cytokines, such as TNF-*α*, interleukin-1*β*, and interleukin-6, further damaging pancreatic β-cell function and exacerbating IR. This creates a vicious cycle involving IR, mitochondrial damage, and inflammation (38). Moreover, IR disrupts energy metabolism in oocytes. Glucose transporter type 4 (GLUT4), which is essential for supplying energy to cells, shows reduced expression in patients with PCOS and IR. This reduction leads to decreased glucose uptake and utilization by granulosa cells, ultimately compromising the oocyte quality (39, 40). Interestingly, Wu et al. demonstrated that knockdown of insulin receptor (INSR) expression in ovarian membrane cells resulted in lower androgen levels and improved fertility in mice (41). Furthermore, IR negatively affects oocyte quality and reduces endometrial receptivity through several mechanisms. These mechanisms include disrupted energy metabolism, altered AMP-activated protein kinase (AMPK) signaling, impaired insulin receptor substrate (IRS)/PI3K/Akt pathways, and ongoing inflammation. Together, these factors contribute to a decline in female reproductive function (42–44).

The EHC is considered the gold standard for assessing metabolic IR *in vivo*. This method measures the impact of insulin on systemic glucose uptake by administering precise insulin infusions and adjusting the glucose infusion rates to maintain normal blood glucose levels. Frequent arterialized glucose measurements and feedback mechanisms are employed throughout this process (45, 46). Owing to the complexity and cost of EHC, there is an increasing need for easily accessible fasting glucose homeostasis parameters as an alternative method for diagnosing IR. Many studies have identified METS-IR as a reliable and practical biomarker for IR, making it a proposed tool for identifying individuals at high risk of T2DM and cardiovascular diseases (CVDs) at an early stage (22, 47). Recently, Xie et al. (15) studied the relationship between various IR surrogates (HOMA-IR, TyG, and TyG-BMI indices) and female infertility. They discovered that higher TyG-BMI levels were positively associated with infertility among reproductive-aged females in the U.S. However, the relationship between METS-IR and infertility remains unclear.

This study explored the relationship between METS-IR and female infertility using data from NHANES. Our findings indicate that elevated METS-IR levels are associated with an increased likelihood of infertility in women aged 18–45 years. Descriptive analysis showed that the mean METS-IR values were significantly higher in the infertility group than in the non-infertility group (Supplementary Table S1), suggesting that METS-IR could predict fertility status. When we categorized the participants into METS-IR tertiles, we observed a noticeable increase in the mean fasting blood glucose and infertility percentages from tertiles 1 to 3 (Table 1). Additionally, we found that the infertility percentage increased with increasing METS-IR among Mexican Americans and Non-Hispanic Black individuals (Table 1). Furthermore, we investigated the METS-IR and infertility relationships using weighted multivariate logistic regression models. As expected, viewing METS-IR as a categorical variable was a more informative independent risk factor for infertility than treating it as a continuous variable (Table 2). Subsequent RCS analysis and threshold effect analysis revealed a linear positive association between METS-IR and infertility (Figure 2 and Table 3). Significantly, the risk of infertility increased with higher METS-IR levels, notably when exceeding the threshold of 32.94 (*p* < 0.0001). These results suggest that METS-IR is particularly predictive of infertility risk in cases of insulin resistance and dyslipidemia. This finding is consistent with previous research showing that women with metabolic syndrome have a negative effect on fertility (48).

Female infertility is influenced by many factors, including age, lifestyle, metabolism, genetics, psychological and occupational stress, as well as experiences of familial abuse and socioeconomic status (49–52). This study examined whether METS-IR is a potentially effective indicator of the relationship between insulin resistance and infertility. Our findings highlight the importance of the METS-IR index as an effective tool for identifying women at an increased risk of infertility. However, this study has certain limitations. First, owing to the constraints of the NHANES database, the definition of female infertility as an outcome variable relied on self-reported data. Self-reports are a commonly used measurement method but may lack precision in specific contexts. For instance, the sample may have included women who had been trying to conceive for less than a year but had already sought medical assistance. Additionally, inadequate management of reproductive health can lead to ovulatory disorders, irregular menstrual cycles, or undiagnosed metabolic conditions, such as PCOS and insulin resistance, which can increase the risk of infertility. This study relied on self-reported data, which may not have fully reflected these issues. It is essential to acknowledge that the frequency of sexual intercourse contributes significantly to female infertility. Couples may face reduced chances of conception if they do not live together, have infrequent intercourse, or engage in intercourse only during nonovulatory periods. Furthermore, women with a family history of infertility may find it more challenging to conceive than those without such a background. Additionally, varying definitions of infertility, such as those based on medical records or time-to-conception derived from calendars, can influence the estimated prevalence (53, 54). Future studies should address the potential impacts of these definitions. It is important to note that this study relied on epidemiological survey data and did not include direct clinical validation, which may have impacted the accuracy and rigor of the findings. Second, as this was a cross-sectional study, we did not include a comparison group of age- and ethnicity-matched fertile women, which prevented us from establishing causal relationships. Finally, because this study focused exclusively on the U.S. population, it remains unclear whether our findings can be generalized to other countries or ethnicities, warranting further investigation.

## Conclusion

In conclusion, this study indicated a positive linear relationship between METS-IR and the likelihood of infertility in females. Given the detrimental effect of IR on women’s fertility, METS-IR could serve as an effective tool for identifying high-risk individuals.

## Data Availability

The datasets presented in this study can be found in online repositories. The names of the repository/repositories and accession number(s) can be found at: https://wwwn.cdc.gov/nchs/nhanes/Default.aspx.
