# Supplementary material for: Association between METS-IR and female infertility: a cross-sectional study of NHANES 2013–2018
Source: Front Nutr. 2025 Feb 28;12:1549525. doi: 10.3389/fnut.2025.1549525 (PMC11906314; doi:10.3389/fnut.2025.1549525)
Supplement: Supplementary file 1 [file Table_1.docx]

| **Table S1 Basic characteristics of the participants*.** |
| --- |

|  | Non-infertility  (n=1353, 87.80%) | Infertility  (n=188, 12.20%) | P value |
| --- | --- | --- | --- |
| **Age(years)** | 30.85 (30.34,31.36) | 34.86 (33.58,36.13) | <0.0001 |
| **Race (%)** |  |  | 0.0694 |
| Mexican American | 12.34 (9.50,15.87) | 9.27 (5.42,15.38) |  |
| Other Hispanic | 8.15 (6.35,10.40) | 5.54 (2.67,11.15) |  |
| Non-Hispanic White | 54.76 (49.93,59.51) | 64.60 (56.13,72.25) |  |
| Non-Hispanic Black | 13.33 (10.54,16.72) | 11.11 (8.03,15.17) |  |
| Other Race - Including Multi-Racial | 11.42 (9.54,13.62) | 9.48 (5.82,15.07) |  |
| **Marital status (%)** |  |  | <0.0001 |
| Married | 43.86 (40.29,47.49) | 69.27 (61.15,76.35) |  |
| Widowed | 0.34 (0.13,0.86) | 0.31 (0.04,2.25) |  |
| Divorced | 6.72 (4.91,9.12) | 7.94 (4.31,14.17) |  |
| Separated | 2.95 (2.20,3.94) | 1.64 (0.61,4.32) |  |
| Never married | 31.19 (27.44,35.21) | 8.97 (5.84,13.52) |  |
| Living with partner | 14.94 (12.78,17.40) | 11.87 (7.15,19.08) |  |
| **Education level (%)** |  |  | 0.1169 |
| Less than high school | 13.55 (11.20,16.30) | 12.55 (8.75,17.70) |  |
| High school or equivalent | 22.60 (19.17,26.44) | 16.39 (10.81,24.08) |  |
| College or above | 63.85 (59.29,68.17) | 71.05 (63.45,77.63) |  |
| **Fasting blood glucose (mg/dL)** | 97.67 (96.35,98.98) | 102.38 (97.80,106.96) | 0.0865 |
| **HDL (mg/dL)** | 58.15 (56.89 ,59.42) | 55.12 (51.89 ,58.35) | 0.0868 |
| **Triglyceride (mg/dL)** | 92.78 (85.57 ,99.98) | 97.85 (84.73 ,110.97) | 0.5016 |
| **Family PIR** | 2.51 (2.38,2.65) | 2.97 (2.53,3.42) | 0.0675 |
| **Smoking status (%)** |  |  | 0.2759 |
| Yes | 31.07 (27.15,35.28) | 35.49 (28.10,43.65) |  |
| No | 68.93 (64.72,72.85) | 64.51 (56.35,71.90) |  |
| **Drinking status (%)** |  |  | 0.0120 |
| Yes | 6.05 (4.93,7.41) | 12.93 (7.14,22.30) |  |
| No | 93.95 (92.59,95.07) | 87.07 (77.70,92.86) |  |
| **Hypertension (%)** |  |  | <0.0001 |
| Yes | 13.32 (11.01,16.02) | 29.29 (21.41,38.64) |  |
| No | 86.68 (83.98,88.99) | 70.71 (61.36,78.59) |  |
| **Diabetes (%)** |  |  | 0.0101 |
| Yes | 4.79 (3.59,6.38) | 10.25 (6.30,16.25) |  |
| No | 95.21 (93.62,96.41) | 89.75 (83.75,93.70) |  |
| **Dyslipidemia (%)** |  |  | 0.0045 |
| Yes | 14.64 (12.69,16.83) | 24.82 (17.62,33.74) |  |
| No | 85.36 (83.17,87.31) | 75.18 (66.26,82.38) |  |
| **Menarche (years)** | 12.56 (12.45,12.66) | 12.48 (12.15,12.80) | 0.6301 |
| **PID (%)** |  |  | 0.0008 |
| Yes | 3.64 (2.53,5.21) | 8.14 (4.59,14.01) |  |
| No | 96.36 (94.79,97.47) | 91.86 (85.99,95.41) |  |
| **Birth control pills (%)** |  |  | 0.0339 |
| Yes | 72.28 (69.40,74.98) | 80.41 (72.94,86.21) |  |
| No | 27.72 (25.02,30.60) | 19.59 (13.79,27.06) |  |
| **Female hormones (%)** |  |  | 0.1177 |
| Yes | 4.31 (3.17,5.83) | 7.78 (3.90,14.92) |  |
| No | 95.69 (94.17,96.83) | 92.22 (85.08,96.10) |  |
| **BMI** | 28.99 (28.41,29.58) | 32.11 (30.50,33.72) | 0.0007 |
| **METS-IR** | 41.22(40.24 ,42.20) | 46.87(44.01,49.73) | 0.0005 |

BMI, body mass index; PIR, poverty impact ratio; PID, pelvic infection/inflammatory disease; METS-IR, metabolic score for insulin resistance.

^*^Percentage estimates are nationally representative using survey weights.
